# Multi-Mycotoxin Occurrence and Health Risk Assessment in Early Post-Harvest Durum Wheat, Barley, and Maize from Tunisia

**DOI:** 10.3390/toxins18050230

**Published:** 2026-05-16

**Authors:** Khouloud Ben Hassouna, Jalila Ben Salah-Abbès, Samir Abbès, Ferjeni Zouidi, Mourad Jridi, Albert Sebastià, Noelia Pallarés, Houda Berrada

**Affiliations:** 1 Laboratory of Analysis, Treatment and Valorization of Environmental Pollutants and Products, Faculty of Pharmacy, Monastir University, Monastir 5000, Tunisia; khouloudhsn20@gmail.com (K.B.H.); jalila.bensalah@yahoo.fr (J.B.S.-A.); 2 Laboratory of Genetic, Biodiversity and Bio-Resources Valorisation, Higher Institute of Biotechnology of Monastir, University of Monastir, Monastir 5000, Tunisia; 3 Al-Ayen University (AUIQ), Nile St., Nasiriyah 64001, Dhi Qar, Iraq; 4 Department of Biology, Faculty of Science, King Khalid University, Abha 62521, Saudi Arabia; zouidiferjeni70@gmail.com; 5 High Institute of Biotechnology of Béja, University of Jendouba, Jendouba 8189, Tunisia; jridimourad@gmail.com; 6 Preventive Medicine and Public Health, Food Science, Toxicology and Forensic Medicine Department, Faculty of Pharmacy, Universitat de València, Burjassot, 46100 València, Spain; albert.sebastia@uv.es (A.S.); houda.berrada@uv.es (H.B.)

**Keywords:** mycotoxins, cereals, Tunisia, UHPLC–MS/MS, risk assessment

## Abstract

The contamination of cereals by mycotoxins represents a major concern due to their harmful effects on human health and food quality. The current study investigated the occurrence of major mycotoxins (AFB1, AFB2, AFG1, AFG2, OTA, ENA, ENA1, ENB, and ENB1) in 158 raw cereal samples (durum wheat, barley, and maize) collected from two Tunisian regions: Beja (continental region) and Mahdia (coastal region). Mycotoxins were extracted using the QuEChERS method and quantified by UHPLC–MS/MS. Several mycotoxins were detected at high levels across all the cereals. In the Beja region, durum wheat was contaminated with AFB1, AFG1, ENA, ENA1, ENB, and ENB1, with ENB being the most frequent (70%). Mahdia durum wheat was contaminated only with ENA, ENA1, ENB, and ENB1, with ENB1 being the most prevalent (22.6%). Barley from both regions was contaminated only with ENs. The ENB was the most frequent (Beja 66%, Mahdia 28.6%). Maize from Beja was contaminated by OTA and ENs, with ENA1 being the most frequent (22.5%), while maize from Mahdia was contaminated by AFB1, AFB2, AFG2, and ENs (AFB1 was the most frequent, 35%). All wheat samples contaminated with AFG1 (6.55%) exceeded the European Union maximum limit for AFs in cereals (4 µg/kg). Similarly, maize samples contaminated with AFB1 (17.5%), AFB2 (10%), and AFG2 (2.5%) exceeded the EU maximum limit for aflatoxins in maize (10 µg/kg). Additionally, maize samples contaminated with OTA (5%) exceeded the EU maximum limit for OTA in unprocessed cereals (5 µg/kg). The co-occurrence of multiple mycotoxins was observed in all cereal types, with up to six different mycotoxins detected in a single sample. Dietary risk assessment revealed high EDIs of AFB1, AFG1, and ENs through the consumption of wheat and barley by Tunisian adults. The calculated MOE values for AFB1 and AFG1 in wheat were below 10,000 (MOE = 1190 for AFB1 and 2.5 for AFG1), suggesting a potential health concern associated with dietary exposure. Despite this potential risk, AFB1 and AFG1 were detected in only 3% and 7% of the analyzed samples, respectively. These results highlight the need for regular monitoring and the establishment of regulations to control mycotoxins in Tunisian cereals.

## 1. Introduction

Cereal grains, including wheat, maize, and barley, are the world’s most important base food [1]. Cereal grains have a high-quality content of carbohydrates, proteins, and dietary starch; they are composed of approximately 75% carbohydrates, essentially starches, and about 6 to 15 percent proteins, and they contribute more than 50% of the energy source [2]. They have other interesting nutrients like fatty acids, vitamins (vitamins E and B), and minerals (calcium, magnesium, potassium, phosphorus, iron, and sodium) [2,3]. In addition, the healthy eating pyramid is based on high-quality cereals, which play an important role in both human nutrition and animal feed preparation (feed meals, concentrates, and silages) [4]. Cereals cover more than 73% of the total harvested area and account for more than 60% of global food production [5]. Its production in 2022 was expected to be 2799 million tons worldwide [6]. Tunisia is one of the world’s top cereal producers, with a production of 1.807 million tons in 2022, and durum wheat is the most prevalent cereal produced (1.250 million tons) [7]. Wheat is primarily grown in monoculture systems, particularly in Tunisia’s north and northwest, especially in the Beja province, and is known as a nitrogen-demanding agrosystem [8]. Cereals are a prevalent and frequently eaten food in Tunisia. Wheat, barley, and maize are staple foods for Tunisians that can be eaten as unprocessed grains, flours, or processed products (bread, besissa, breakfast cereals, couscous, pasta, cakes, corn oil, corn syrup, cornmeal, malt, soup, etc.) [9]. Good quality control of cereals and their derivatives is essential, with special attention given to the field cultivation phase as the first step in determining their quality. Unfortunately, climate change causes global warming, with the Earth’s temperature expected to increase by 1.5 to 4.5 degrees Celsius by the end of the twenty-first century, potentially affecting crop productivity [10]. Tunisia, as a North African country, is known for its Mediterranean climate, which is warm and humid [9]. These conditions favor toxigenic mold growth, which ends up in the production of secondary metabolites in foods [11]. Those secondary metabolites are named mycotoxins, and they are mainly produced by the genera *Aspergillus*, *Alternaria*, *Claviceps*, *Fusarium*, and *Penicillium* [9]. Nowadays, more than 500 mycotoxins have been discovered worldwide [9]; however, aflatoxins (AFs), ochratoxin A (OTA), zearalenone (ZEN), fumonisins (FMs), patulin (PAT), and trichothecenes (TRC), including deoxynivalenol (DON) and T-2 toxin (T-2), are the most frequently detected in food, particularly cereals [12,13]. In response to this global concern, the European Union [14] has established maximum concentrations for those mycotoxins in cereals meant for human consumption. The recommended limits for mycotoxins in cereals other than maize are 4 μg/kg, 2 μg/kg, 5 μg/kg, and 100 μg/kg for AFs, AFB1, OTA, and ZEN, respectively [14]. Concerning maize, the established concentrations are 350, 5, 2, and 10 μg/kg for ZEN, OTA, AFB1, and AFs, respectively [14]. Furthermore, Tunisia established a maximum permitted level (MPL) of 2 µg/kg for AFB1 in cereals and its products [15]. By controlling and limiting the amount of these mycotoxins present in grains, these regulations aim to protect the public’s health. However, in addition to those regulated mycotoxins, *Fusarium* spp. produce other mycotoxin kinds known as “emerging mycotoxins”, which are not regulated due to their limited toxicological information [16]. This group of mycotoxins is mostly composed of enniatins (ENs), beauvericin, and moniliformin [17]. In fact, four analogs, enniatin A (ENA), enniatin A1 (ENA1), enniatin B (ENB), and enniatin B1 (ENB1), are the most often found among the 29 known ENs in cereal grains [18]. The principal ENs-producing species include *Fusarium avenaceum* and *Fusarium tricinctum*, which are considered the major producers of ENA, ENA1, ENB and ENB1. Other species, such as *Fusarium equiseti*, *Fusarium acuminatum* and *Fusarium lateritium*, have also been reported to produce ENs in cereals [19]. According to Eskola et al. [20], mycotoxins infect 25% of grains consumed worldwide, and this finding could be even higher. Mycotoxins can contaminate cereals before or after harvest and can be caused by a variety of factors, such as humidity, temperature, inappropriate storage conditions, insect damage, processing conditions, etc. [21]. As mycotoxins are chemically very stable and do not degrade during food processing, they induce a wide range of severe and hazardous health effects, such as cytotoxicity, hepatotoxicity, immunosuppression, genotoxicity, nephrotoxicity, and even carcinogenicity [13,17,22]. Fortunately, the key worldwide initiatives for mycotoxin control in wheat and other small grains were recently evaluated [23]. In fact, for multi-mycotoxin analysis, a quick, easy, cheap, rugged, effective, and safe (QuEChERS) extraction technique has been employed recently. Additionally, due to their excellent selectivity, precision, and sensitivity, high-performance liquid chromatography (HPLC) coupled to a fluorescence detector or tandem mass spectrometry (MS) has been employed experimentally to determine mycotoxins; ultrahigh pressure (UP) coupled to MS has also been used [9,24,25].

Even if cereals are consumed in large quantities in Tunisia, there is a clear lack of studies concerning the mycotoxin contamination of these important foods and their risk assessment [9,26,27,28]. In particular, data on maize contamination are still scarce, and the Mahdia region has received limited attention compared to other cereal-producing regions. In addition, few Tunisian studies have compared regional differences in mycotoxin occurrence while combining contamination data with dietary exposure and risk assessment. Therefore, further investigation is needed to improve knowledge on the distribution of mycotoxins in Tunisian cereals and their potential health risks. Taking those considerations into account, the current study aims to determine multi-mycotoxin occurrence in Tunisian cereals (durum wheat, barley, and maize) collected from two different regions (Beja, a continental region, and Mahdia, a coastal region) by the use of the UHPLC-MS/MS, as well as the Tunisian population risk assessment, which has been calculated.

## 2. Results and Discussion

Durum wheat, barley, and maize are among the most widely consumed cereals in North Africa, particularly in Tunisia [29]. Due to their important role in the local diet, the safety and quality of these cereals are a major concern. In this study, the occurrence of both regulated and emerging mycotoxins was investigated in cereal samples collected from two distinct regions of Tunisia: Beja, representing a continental region, and Mahdia, representing a coastal region.

### 2.1. Occurrence of Mycotoxins in Tunisian Cereals

A total of 158 Tunisian raw cereal grain samples, including 61 durum wheat, 57 barley, and 40 maize samples, were analyzed using UHPLC-MS/MS to assess the prevalence of both regulated and emerging mycotoxins. The regulated mycotoxins included aflatoxins (AFB1, AFB2, AFG1, and AFG2) and ochratoxin A (OTA), while the emerging *Fusarium* mycotoxins included enniatins (ENA, ENA1, ENB, ENB1). Table 1 summarizes the occurrence, minimum and maximum concentrations, mean concentration in positive samples, and mean concentration across all samples for each detected mycotoxin in the different cereal types. Only positive samples were considered for calculations. A sample was considered positive when the mycotoxin concentration exceeded the limit of detection (LOD).

The analysis of durum wheat samples revealed contamination by AFB1, AFG1 and ENs (ENA, ENA1, ENB and ENB1), with ENs showing a higher detection frequency than the regulated aflatoxins. AFB1 was detected in only 2 out of 61 samples, but at levels below the LOQ. In contrast, AFG1 was detected in four samples, with relatively high concentrations ranging from 100.04 to 127.75 µg/kg. These findings highlight a notable presence of AFG1 in durum wheat, while AFB1 occurrence remains limited. Aloui et al. [27] also reported the occurrence of AFB1 in 13% of Tunisian durum wheat samples. In contrast, Oueslati et al. [30] did not detect AFB1 in Tunisian wheat samples, suggesting that mycotoxin occurrence can vary considerably depending on the harvest year and environmental conditions. Studies conducted in other North African countries have also reported the presence of AFB1 and AFG1 in wheat samples. For example, AFB1 was detected in wheat from Algeria and Egypt, with occurrence rates of 60.7% and 33%, respectively, which are higher than those observed in the present study [31,32]. AFG2 was not detected in the present study, although its presence at high concentrations has been reported in wheat samples in other Tunisian studies [28,30]. AFB2 was not detected in our samples, which is consistent with the findings of Oueslati et al. [33] in Tunisian wheat. OTA was not detected in our durum wheat samples. This finding aligns with the results of Serrano et al. [34], who also reported the absence of OTA in Tunisian wheat. However, Jedidi et al. [35] detected OTA in one wheat sample from Tunisia at a concentration of 3.30 µg/kg. Regarding ZEN, Zaied et al. [36] reported its presence in 80% of durum wheat samples, with a mean concentration of 58 µg/kg. Additionally, Jedidi et al. [37] identified ZEN in two samples at concentrations of 21.5 and 32.4 µg/kg, respectively. ENs were detected in raw durum wheat samples at relatively high concentrations. ENB was the most frequently detected, found in 45.9% of the samples, with concentrations ranging from 0.44 to 1385.83 µg/kg, with the highest maximum level recorded among all ENs. ENB1 was the second most detected (32.78%), with concentrations ranging from 15.06 to 155.81 µg/kg. ENA1 was detected in 31.1% of the samples, with levels ranging from 45.63 to 350.33 µg/kg. In contrast, ENA was the least frequently detected, found in 16.4% of the samples, at concentrations ranging from 60.53 to 131.75 µg/kg. Several studies have also reported the presence of ENs in Tunisian wheat. Oueslati et al. [38] detected ENA, ENA1, ENB, and ENB1 in all raw wheat samples analyzed, with average concentrations of 90,000 µg/kg for ENA, 75,000 µg/kg for ENA1, 30,000 µg/kg for ENB, and 48,000 µg/kg for ENB1. These concentrations were higher than those observed in the present study. More recently, Aloui et al. [27] reported the presence of ENA1, ENB, and ENB1 in 60.9% of wheat samples. In line with our findings, their results also indicated that ENB was the most commonly detected, present in 54.3% of samples. However, the concentrations they reported were considerably higher, ranging from 30 to 7684 µg/kg for ENB, 48 to 1208.4 µg/kg for ENB1, and 42 to 1410 µg/kg for ENA1. In another study, Oueslati et al. [33] reported higher concentrations of ENs in processed foods, with levels of 54.5 µg/kg for ENA, 78.2 µg/kg for ENA1, 51.5 µg/kg for ENB, and 30.6 µg/kg for ENB1, compared with the lower levels observed in the raw wheat samples analyzed in this study. In contrast to wheat samples, barley was contaminated exclusively with emerging *Fusarium* mycotoxins, namely ENA, ENA1, ENB, and ENB1. Regulated mycotoxins, including AFs, OTA, and ZEN, were not detected. ENB was the most frequently detected mycotoxin (47.37%), with concentrations ranging from 16.75 to 266.14 µg/kg. ENB1 was detected in 15.79% of samples, at concentrations ranging from 12.33 to 79.17 µg/kg. ENA1 was detected in 12.28% of samples, with concentrations ranging between 46.16 and 167.52 µg/kg. ENA was the least frequently detected (7%), with concentrations ranging from 60.98 to 81.66 µg/kg. Our findings are consistent with those of Juan et al. [39], who also detected ENs in Tunisian barley. However, the contamination profiles differed. In their study, ENA was detected in 16% of samples at concentrations ranging from 31.6 to 287.1 µg/kg, while ENA1 was found in 10% of samples (40.5–220.3 µg/kg). ENB and ENB1 were each detected in 10% of samples, with concentrations ranging from 107.3 to 184.6 µg/kg and from 112.4 to 162.7 µg/kg, respectively. The overall concentrations reported were generally higher than those observed in the present work. Oueslati et al. [38] also reported the presence of ENs in 80% of Tunisian barley samples, with considerably higher mean concentrations (33,600 µg/kg for ENA, 116,400 µg/kg for ENA1, 27,500 µg/kg for ENB, and 31,000 µg/kg for ENB1). The occurrence of ENs in barley has been investigated in studies from other North African countries. Zinedine et al. [40] reported the presence of ENs, except ENA, in Moroccan barley. The mean concentrations were 84,000 µg/kg for ENB, 18,000 µg/kg for ENB1, and 22,000 µg/kg for ENA1, which were higher than those observed in the present study. In contrast, Mahdjoubi et al. [41] did not report the detection of ENs in barley samples from Algeria, suggesting possible regional variability in *Fusarium* contamination. Neither aflatoxins nor ochratoxin A was detected in the barley samples analyzed in the present study. This finding is consistent with the results of Oueslati et al. [30], who also reported the absence of OTA in barley samples collected in 2009. In contrast, Juan et al. [39] reported the presence of OTA in 6% of barley samples, although at very low mean concentrations (0.0006 µg/kg). ZEN was not analyzed in any of the barley samples in this study, whereas Jedidi et al. [37] identified ZEN in a single barley sample at a concentration of 21.2 µg/kg.

Maize samples were contaminated with both regulated mycotoxins (OTA, AFB1, AFB2, and AFG2) and emerging mycotoxins (ENA, ENA1, ENB, and ENB1). Among the regulated mycotoxins, AFB1 was the most frequently detected, present in 7 out of 40 maize samples (17.5%). AFB2 was detected in 4 samples (10%), OTA in 2 samples (5%), and AFG2 in only one sample (2.5%). In contrast to our findings, Oueslati et al. [33] did not find OTA, AFB1, AFB2, or AFG2 in Tunisian maize, detecting only AFG1 in one sample. AFB2 was the most abundant mycotoxin in the analyzed maize samples, with concentrations ranging from 16.89 to 2236.82 µg/kg and a mean of 115.04 µg/kg. AFB1 was also present at high levels, with concentrations ranging between 40.94 and 1769.89 µg/kg and a mean of 261.04 µg/kg. AFG2 was detected in only one sample, but at a notably high concentration of 593.17 µg/kg. In line with our findings, Jedidi et al. [37] reported high AF levels in Tunisian maize, ranging from 1.13 to 99.32 µg/kg in post-harvest samples and 1.00 to 969.18 µg/kg in freshly harvested maize. Abdallah et al. [42] also detected AFB1 and AFB2 in maize from Egypt, with concentrations of 0.2–44.9 µg/kg and 0.1–7.0 µg/kg, respectively; however, these levels were considerably lower than those observed in our study. OTA was detected in only two maize samples in our study, but at relatively high concentrations (92.88–99.93 µg/kg). In contrast, Mahdjoubi et al. [41] did not detect OTA in any maize samples in Algeria, a North African country with climatic conditions similar to Tunisia. Concerning ENs, maize was mainly contaminated with ENA and ENA1, each detected in 22.5% of samples. These ENs exhibited the highest mean concentrations of 60.88 µg/kg and 45.47 µg/kg, respectively. ENB and ENB1 were less common, found in 17.5% and 10% of samples, with lower mean levels of 17.46 µg/kg and 15.16 µg/kg. This study represents, to the best of our knowledge, the second report of EN contamination in raw maize in Tunisia. In the other study, Oueslati et al. [38] detected only ENA1 in raw maize from Tunisia, present in 2 of 3 samples (66.7%). Mahdjoubi et al. [41] reported both ENA1 and ENB1 in Algerian maize, with mean concentrations of 56.4 µg/kg and 60.9 µg/kg, respectively, which were substantially higher than the levels observed in our samples. To date, information on the presence of toxigenic fungi and their associated mycotoxins in maize grains in Tunisia remains limited. Therefore, there is an urgent need for comprehensive surveys to better evaluate fungal contamination and associated mycotoxin profiles in maize. The high prevalence of emerging mycotoxins observed in Tunisian cereals in this study may be linked to the presence of EN-producing *Fusarium* species, such as *Fusarium equiseti*, which have been detected in Tunisian wheat and other cereals [43].

In wheat, all samples contaminated with AFG1 (6.55%) from the two studied regions showed concentrations exceeding the maximum limit for AFs in cereals and cereal products (4 µg/kg) established by the European Union. Similarly, maize samples from the two regions contaminated with AFB1 (17.5%), AFB2 (10%), and AFG2 (2.5%) also presented levels above the EU regulatory limit for aflatoxins in maize (10 µg/kg). In addition, OTA detected in maize samples (5%) exceeded the maximum limit set by the European Union for OTA in unprocessed cereals (5 µg/kg). In the present study, the contamination of Tunisian cereal samples by mycotoxins and the relatively high concentrations observed may be influenced by several agronomic and environmental factors, such as the use of susceptible cereal varieties, crop rotation practices, and reduced or no-tillage systems. However, these factors were not assessed in this study; therefore, this interpretation remains hypothetical. Since the samples were collected during the early post-harvest period, the results suggest that contamination may have occurred either pre- or post-harvest, highlighting the potential importance of field and storage conditions in fungal development and mycotoxin production. Further studies are required to identify the specific factors contributing to contamination under Tunisian conditions.

### 2.2. Regional Variations in Mycotoxin Contamination Levels

The occurrence and distribution of different mycotoxins in cereal samples collected from Beja (a continental region) and Mahdia (a coastal region) in Tunisia are presented in Table 2. The mycotoxin profile in each region was described based on detection frequency and concentration levels. For each mycotoxin, the minimum, maximum, and mean concentrations were calculated for positive samples as well as for all analyzed samples. Only positive samples were considered for calculations. A sample was considered positive when the mycotoxin concentration exceeded the limit of detection (LOD).

The results showed clear regional differences in mycotoxin contamination among the different cereal types. AFB1 contamination showed a clear dependence on both crop type and regional agro-climatic conditions. In fact, AFB1 was detected exclusively in durum wheat from the continental region, with a low incidence of 7% and concentrations below the LOQ. In another Tunisian study, Aloui et al. [27] examined mycotoxin contamination in durum wheat collected from the same continental region (Beja) as the present investigation, as well as from a coastal region (Kairouan). Their findings align with the observations reported in this study, showing that AFB1 was detected exclusively in wheat from the continental region, but with a higher incidence of 30% and concentrations between 17 and 37.8 µg/kg. AFB1 was not detected in maize from the continental region (Beja). Instead, it occurred only in maize samples from the coastal region (Mahdia), where it was present in 35% of samples and reached much higher concentrations (40.94–1769.89 µg/kg). AFB1 was also reported in sorghum collected from another Tunisian coastal region (Sousse), geographically close to the sampled region in this work, with an average concentration of 1.24 µg/kg [44]. AFB2 was not detected in any samples collected from the continental region. In contrast, it was found in 20% of maize samples from the coastal region, where concentrations ranged from 16.89 to 2236.82 µg/kg. Also, in our previous study, we did not detect AFB2 in wheat samples collected from the same region [28]. AFG1 was detected in 13% of durum wheat samples from the continental region, with levels ranging from 100.44 to 127.55 µg/kg. This mycotoxin was completely absent in all samples from the coastal region, regardless of the cereal type examined. These results suggest that AFG1 may be linked to continental environmental conditions. Findings from Ben Hassouna et al. [28] support part of this pattern, as they also did not detect AFG1 in wheat from the coastal region (Mahdia). However, they also did not report AFG1 in wheat from the continental region (Beja). Such differences may be related to changes in climate, sampling period, or fungal contamination from one year to another. AFG2 was detected in only one maize sample from the coastal region, with a high concentration of 593.17 µg/kg. This mycotoxin was not found in any other cereal type or in any sample from the continental region. These results differ from those of Ben Hassouna et al. [28], as AFG2 was detected in wheat from both the continental (Beja) and coastal (Mahdia) regions in their study, which correspond to the same areas examined in the present work. OTA was detected only in maize samples from the continental region, showing an incidence of 10% and concentrations between 92.88 and 99.93 µg/kg and was absent in other cereal types from this region as well as in all samples from the coastal region. These results partially agree with previous reports. Lahouar et al. [44] detected OTA in sorghum from Sousse, a coastal region near Mahdia, although at much lower concentrations (average 1.71 µg/kg). In contrast, Lasram et al. [45] did not detect OTA in pearl millet collected from Mahdia, which aligns with the absence of this mycotoxin in our samples from Mahdia. Overall, these findings suggest that OTA occurrence may be both crop- and region-specific in Tunisia.

ENA, ENA1, ENB, and ENB1 were detected in durum wheat, barley, and maize from both the continental region (Beja) and the coastal region (Mahdia). However, their levels and distribution differed noticeably between the two regions. In wheat samples, EN contamination was more frequent in those collected from the continental region than in samples from the coastal region. ENB and ENB1 were the most common ENs in wheat samples from Beja, detected in 70% and 47% of samples, respectively. In contrast, samples from Mahdia showed lower frequencies, with ENB detected in 22.6% of samples and ENB1 in 19.4%. Clear differences were also observed in EN concentration levels. Durum wheat samples from Beja showed higher mean concentrations of ENA (20.34 µg/kg) and ENA1 (39.44 µg/kg) compared with samples from Mahdia (3.97 µg/kg for ENA and 9.29 µg/kg for ENA1). ENB reached the highest levels in samples from Beja, with a mean concentration of 138.15 µg/kg, while samples from Mahdia showed much lower levels (3.91 µg/kg). ENB1 also showed a higher mean concentration in samples from Beja (29.82 µg/kg) than in those from Mahdia (3.78 µg/kg). Aloui et al. [27] recently analyzed wheat samples from the same continental region as our study (Beja) and from another coastal region (Kairouan). They detected only ENA1, ENB, and ENB1, with higher frequencies in continental samples, which is consistent with our results. However, the concentrations they reported for all three ENs were much higher than those observed in wheat samples analyzed in this study. More recently, Hassine et al. [46] analyzed durum wheat from Beja and detected ENA1, ENB, and ENB1 at mean concentrations of 1.69, 10.13, and 3.38 µg/kg, respectively, which were lower than those observed in the present study. Chakroun et al. [26] analyzed durum wheat from the same continental region and found only ENA and ENB1, both at extremely high levels compared with our data. In barley samples, the concentration levels of ENs were generally higher in samples from Mahdia. In barley from Beja, the mean concentrations were 2.10 µg/kg for ENA, 4.81 µg/kg for ENA1, 12.36 µg/kg for ENB, and 2.98 µg/kg for ENB1. In comparison, barley samples from Mahdia showed higher mean levels, with 7.66 µg/kg for ENA, 13.61 µg/kg for ENA1, 17.60 µg/kg for ENB, and 5.01 µg/kg for ENB1. To date, no studies have been conducted in Tunisia on the occurrence of ENs in barley from Beja, Mahdia, or other similar regions.

The high frequencies and concentrations of mycotoxins observed in maize from the coastal region (Mahdia) may be due to regional variations in environmental conditions. Maize from Mahdia was contaminated by a greater diversity of mycotoxins than maize from the continental region (Beja), reaching up to 2236.82 µg/kg for AFB2 and 1113.37 µg/kg for ENB. Indeed, as shown in Table 3, the coastal region had higher humidity (66%) compared to the continental region (58%) during the maize sampling period (July–August). Higher humidity in coastal regions can favor fungal growth, which likely explains the elevated mycotoxin frequencies and concentrations in maize from Mahdia.

In wheat samples from the continental region (Beja), high prevalence and frequencies of ENs, reaching 1385.83 µg/kg for ENB, were reported, and this can be attributed to differences in climatic conditions between the two regions. According to Table 3, the agro-climatic conditions in Beja, characterized by slightly higher temperatures (32.5 °C) compared to Mahdia (30 °C) during wheat collection (June–July), may favor fungal growth and subsequent ENs production in wheat grains. In barley, EN frequencies were also higher in samples from Beja than in those from Mahdia; however, the concentrations were higher in Mahdia samples, reaching 266.14 µg/kg for ENB. This can be explained by the higher humidity in Mahdia (62%) compared to Beja (50%) during barley sampling (June–July). These observations suggest that barley is more sensitive to humidity, whereas wheat is more influenced by temperature in relation to EN contamination. In contrast to wheat and barley, EN contamination in maize was higher in samples from the coastal region (Mahdia) than in those from the continental region (Beja). In Mahdia, the detection frequencies ranged from 15% to 35%, and the contamination levels were notably higher. Mean concentrations were 23.94 µg/kg for ENA, 14.55 µg/kg for ENA1, 59.23 µg/kg for ENB, and 2.51 µg/kg for ENB1, with ENB reaching a maximum concentration of 1113.37 µg/kg. Maize samples from Beja showed lower frequencies between 5% and 15% and lower mean concentrations of 6.16 µg/kg for ENA, 6.97 µg/kg for ENA1, 1.81 µg/kg for ENB, and 0.76 µg/kg for ENB1.

Regional differences in mycotoxin occurrence in different cereal types may also be related to variations in agricultural and post-harvest practices between the studied regions. In Tunisia, farming practices can differ from one region to another, including crop rotation systems, irrigation intensity, and the use of fertilizers or fungicides, which may influence fungal development in cereals. Differences in cereal varieties grown in each region may also contribute to the observed variability, as some varieties are more susceptible to fungal infection and mycotoxin production than others. In addition, regional differences in harvesting time, grain drying, and storage practices can affect moisture levels in cereals and, consequently, mycotoxin occurrence.

### 2.3. Natural Co-Occurrence of Regulated and Emerging Mycotoxins in the Analyzed Cereal Samples

The co-occurrence of mycotoxins in cereal samples from two Tunisian regions was observed in this study (Table 4).

The results revealed significant multi-contamination, with up to six different mycotoxins detected in a single sample across both regions and cereal types. The identified compounds included regulated mycotoxins found in combinations such as AFB1 + AFG1. The co-occurrence of emerging mycotoxins was also observed, either alone (e.g., ENB + ENB1 or ENA + ENA1 + ENB + ENB1) or in combination with regulated mycotoxins (e.g., AFB1 + AFB2 + ENA + ENA1 + ENB, or ENA + ENA1 + OTA). Wheat samples showed the highest diversity and frequency of mycotoxin co-occurrence among the analyzed cereals. In the continental region (Beja), wheat samples contained from two to six mycotoxins, mainly ENs and AFs. The most common combinations were ENA1 + ENB + ENB1 and ENA + ENA1 + ENB + ENB1. Two samples were contaminated by both ENs and AFs, one with five mycotoxins (AFG1 + ENA + ENA1 + ENB + ENB1) and another with six (AFB1 + AFG1 + ENA + ENA1 + ENB + ENB1). In contrast, wheat samples from the coastal region (Mahdia) contained fewer co-occurring mycotoxins, with either three or four ENS (e.g., ENA1 + ENB + ENB1 or ENA + ENA1 + ENB + ENB1). Aloui et al. [27] also reported the co-occurrence of two to six mycotoxins in Tunisian wheat. In their study, similarly to our findings, multi-mycotoxin contamination in wheat samples collected in 2020 was more frequent in the same continental region (Beja), with samples containing either both aflatoxins (AFs) and ENs or ENs alone. The simultaneous presence of three ENs (ENA1 + ENB + ENB1) in wheat samples from both the continental and coastal regions was also observed, in accordance with our results. Similarly to our results, Blesa et al. [47] reported the co-occurrence of two to six mycotoxins in Moroccan wheat, with several samples simultaneously contaminated by all four ENs. Unlike in Tunisia and Morocco, Mahdjoubi et al. [41] did not observe simultaneous contamination by ENs and AFs in Algerian wheat, suggesting country differences in fungal species distribution and environmental factors influencing mycotoxin production. In this study, the absence of co-occurrence among regulated mycotoxins in wheat samples contrasts with previous Tunisian studies. Ben Hassouna et al. [28] identified the co-contamination with AFG2 and OTA in 17.3% of positive samples from the continental region (Beja), while Hassine et al. [46] also detected the co-occurrence of regulated mycotoxins such as ZEN + OTA + DON and ZEN + OTA in wheat from the same region.

Barley samples exhibited moderate levels of mycotoxin co-occurrence, typically involving two to four mycotoxins, with ENs being the most prevalent. In samples from the continental region (Beja), binary co-occurrence was limited to ENB + ENB1 in 2 samples (6.09%), ternary co-occurrence to ENA1 + ENB + ENB1 in 2 samples (6.09%), and quaternary co-occurrence to ENA + ENA1 + ENB + ENB1 in 1 sample (3.45%), while combinations of five or six mycotoxins were not observed. In contrast, barley from the coastal region (Mahdia) showed a binary co-occurrence of ENB + ENB1 in 1 sample (3.58%) and a higher frequency of quaternary EN co-contamination (ENA + ENA1 + ENB + ENB1) in 3 samples (10.71%), while combinations of three, five, or six mycotoxins were not observed. The co-occurrence of mycotoxins has also been reported in Tunisian barley and derived products. For instance, Oueslati et al. [38] reported the co-occurrence of four ENs (ENA, ENA1, ENB, ENB1) in raw barley, while Juan et al. [39] reported barley contamination with up to four mycotoxins, including regulated toxins such as OTA, ZEN, and DON, in combination with ENs.

Compared with wheat and barley, maize samples exhibited considerably fewer mycotoxin co-occurrences, with most combinations either absent or detected at very low frequencies, indicating that maize is the cereal least affected by multi-mycotoxin contamination. In the continental region (Beja), maize samples showed the presence of three or four mycotoxins, either ENs alone or in combination with AFs. Two samples (10%) presented the co-occurrence of multiple mycotoxins: one sample contained three mycotoxins (ENA + ENA1 + OTA), and one sample contained four mycotoxins (ENA + ENA1 + ENB + ENB1). No samples exhibited the co-occurrence of only two mycotoxins or of five to six mycotoxins. In contrast, maize from the coastal region (Mahdia) displayed a more diverse co-occurrence profile, with two to six mycotoxins detected per sample, either ENs alone or in combination with AFs. Nine samples (45%) showed co-occurrence of two or more mycotoxins. Specifically, five samples (25%) contained two mycotoxins (combinations of AFB2 + AFG2, ENA + ENA1, or ENA + ENB), one sample contained three ENs (ENA + ENA1 + ENB1), one sample contained four ENs (ENA + ENA1 + ENB + ENB1), one sample contained five mycotoxins (AFB1 + AFB2 + ENA + ENA1 + ENB), and one sample contained six mycotoxins (AFB1 + AFB2 + ENA + ENA1 + ENB + ENB1). These results indicate that the co-contamination, involving both *Aspergillus*-derived aflatoxins and *Fusarium*-derived ENs, was more frequent in the coastal region. Previously, Oueslati et al. [38] also reported the co-occurrence of ENs in Tunisian maize, although at higher frequencies than those observed in our study. In their survey, 74% of samples were co-contaminated with at least two ENs, and 11.7% contained the 4 ENs (ENA, ENA1, ENB, and ENB1).

The analysis highlights that coastal regions tend to create more favorable environments for the simultaneous occurrence of multiple mycotoxins in cereal [48]. Overall, the number of co-occurring mycotoxins ranged from two to six per sample. In fact, the co-occurrence of multiple mycotoxins observed in several cereal samples is an important food safety concern. Simultaneous exposure to different mycotoxins may result in additive or synergistic toxic effects, even when individual toxin concentrations are below their regulatory limits. Previous studies have reported that combined exposure may enhance adverse effects such as hepatotoxicity, nephrotoxicity, immunotoxicity, and oxidative stress [49]. Therefore, the presence of multiple mycotoxins in cereals may represent a greater health concern than exposure to individual mycotoxins alone [50].

The co-occurrence of several EN analogs in the same cereal sample can be explained by the ability of the same *Fusarium* isolates to produce multiple structurally related mycotoxins and by the ecological overlap among EN-producing species [51]. In fact, the intrinsic characteristics of cereals, such as grain structure, nutrient content, and moisture retention, can favor the simultaneous colonization of the grain by multiple fungal species [52]. In addition, environmental factors, including temperature and humidity, as well as agronomic and storage practices, can promote fungal growth and lead to the production of multiple mycotoxins in the same sample [28,53]. Although ENs are not yet subject to regulatory limits, their frequent co-occurrence with other mycotoxins raises concerns about possible additive or synergistic toxic effects. Such interactions may increase overall toxicity even when individual compounds remain below established safety thresholds [54]. Previous studies by Alassane-Kpembi et al. [55] and Kifer et al. [56] have demonstrated that these interactions can enhance cytotoxicity in in *vitro* models, including intestinal (Caco-2), hepatic (HepG2), and renal (Vero, PK15, and HK2) cell lines. Consequently, assessing combined exposure to multiple mycotoxins is essential to obtain a more realistic evaluation of the health risks associated with cereal consumption in Tunisia.

These findings highlight the need for cumulative exposure assessment and further studies investigating the combined toxicological effects of co-occurring mycotoxins. In addition, routine multi-mycotoxin monitoring using validated LC–MS/MS methods is essential for the simultaneous detection of ENs and regulated mycotoxins and would provide stronger support for future risk assessment and management strategies.

### 2.4. Assessment of Dietary Exposure to Mycotoxins and Health Risk Characterization

Daily exposure to mycotoxins mainly occurs through the consumption of contaminated cereals and cereal-based products, as these foods are staples in the human diet. Dietary exposure assessment combines data on mycotoxin concentrations in foods with population consumption data, providing essential information for estimating the potential risks associated with mycotoxin intake [57]. In this study, the Estimated Daily Intake (EDI) was calculated for each mycotoxin detected in durum wheat (AFB1, AFG1, ENA, ENA1, ENB, and ENB1) and in barley (ENA, ENA1, ENB, and ENB1). Due to the lack of maize consumption data in Tunisia, the EDI of detected mycotoxins in maize could not be determined. In Tunisia, the average daily consumption is estimated at 716.66 g/day for wheat [58] and 123.33 g/day for barley [59], and the average adult body weight was assumed to be 70 kg. The estimated wheat consumption in Tunisia may appear elevated compared with global averages. However, wheat represents the primary staple food in Tunisia and constitutes the major component of the daily diet, mainly consumed as bread and traditional wheat-based products. Comparable high intake levels of wheat have been reported in other North African countries, including Algeria (502 g/day [41]), the Middle East region (327.3 g/day [31]), and Egypt (537 g/day [60]). In contrast, lower wheat consumption has been documented in Mediterranean European countries such as Italy and Spain. In Italy, the mean daily intake of wheat was 156.7 g/day for males and 121.1 g/day for females [61], while in Spain, the average consumption of wheat was 274.2 g/day [62]. These differences likely reflect variations in dietary patterns, food habits, and the relative contribution of wheat-based products within national diets.

The EDI values provide a basis for assessing potential health risks associated with chronic exposure. The formal health risk characterization associated with dietary intake of ENs was not performed, as no tolerable daily intake (TDI) or other health-based guidance values have yet been established for these compounds. AFB1 and AFG1 are genotoxic and carcinogenic mycotoxins, and no safe intake level can be established; thus, their occurrence in food should be kept as low as reasonably achievable (ALARA). Therefore, risk characterization for AFB1 and AFG1 was performed using the margin of exposure (MOE) approach, which is recommended for substances with carcinogenic potential.

Table 5 presents the mean concentrations of the detected mycotoxins in Tunisian barley and wheat samples collected in 2022, along with their corresponding Estimated Daily Intake (EDI) values and the Margin of Exposure (MOE) calculated for AFB1 and AFG1 in wheat. Regarding EDI values, a difference was observed between dietary exposure to mycotoxins through wheat consumption and that through barley consumption.

In wheat, ENB was the major contributor to dietary exposure, showing the EDI of 716.2 ng/kg bw/day, followed by ENA1 (247.0 ng/kg bw/day), ENB1 (169.8 ng/kg bw/day), AFG1 (161.1 ng/kg bw/day), ENA (123.0 ng/kg bw/day) and AFB1 (0.336 ng/kg bw/day). In contrast, considerably lower EDI values were observed in barley. Among the detected mycotoxins, ENB again showed the highest EDI (26.3 ng/kg bw/day), followed by ENA1 (16.1 ng/kg bw/day), ENA (8.5 ng/kg bw/day), and ENB1 (7.0 ng/kg bw/day). EDI values obtained in the present study indicate that dietary exposure to the detected mycotoxins is notably higher through durum wheat than through barley, with ENB being the principal contributor to intake. The lower EDIs of mycotoxins through the consumption of barley can be explained by both the lower contamination levels and the lower daily consumption of barley in Tunisia. Dietary exposure to mycotoxins through the consumption of wheat, barley, and their derived products has been evaluated in other Tunisian studies. Aloui et al. [27] determined the EDIs of ENA1, ENB, and ENB1 through the consumption of Tunisian durum wheat collected in 2020 and 2021. In samples from 2021, the EDIs of ENA, ENB, and ENB1 were found to be 9.2, 786.6, and 102.4 ng/kg bw/day, respectively. In 2020, the estimated daily intake of ENs was 679.6 ng/kg bw/day for ENA1, 3592 ng/kg bw/day for ENB, and 1363 ng/kg bw/day for ENB1. These EDI values were higher compared with those observed in our study, indicating a greater dietary exposure to ENs through the consumption of wheat in Tunisia. Hassine et al. [46] assessed the EDI of ENs in durum wheat samples collected in Tunisia during 2021 and 2022. In 2021, the EDIs values were 4.30 ng/kg bw/day for ENA1, 105.17 ng/kg bw/day for ENB, and 49.91 ng/kg bw/day for ENB1. In 2022, the EDI values were 18.75 ng/kg bw/day for ENA1, 121.08 ng/kg bw/day for ENB, and 40.92 ng/kg bw/day for ENB1. Overall, these values were lower than the EDIs of ENA, ENB, and ENB1 estimated in the present study. In another study, Juan et al. [39] evaluated the EDIs of ENs from barley and barley-derived products. The EDIs of ENA, ENA1, ENB, and ENB1 through the consumption of barley were 0.99, 0.61, 0.86, and 0.73 ng/kg bw/day, respectively. The EDIs of ENA, ENA1, ENB, and ENB1 through the consumption of barley soup were 0.12, 0.13, 0.28, and 0.30 ng/kg bw/day, respectively. In contrast to our findings, these values indicate a lower dietary exposure to ENs through the consumption of barley. These results indicate that dietary exposure to ENs varies according to the type of barley consumed, with higher intakes associated with raw barley compared to processed products such as soup.

Based on the estimated daily intakes (EDIs) of AFB1 and AFG1, the margin of exposure (MOE) was calculated using a benchmark dose lower confidence limit for a 10% increase in liver cancer incidence (BMDL10) of 0.4 µg/kg bw/day, as established by EFSA for AFB1. Since no compound-specific BMDL10 has been established for AFG1 due to insufficient toxicological data, the same reference value (0.4 µg/kg bw/day) was conservatively applied following EFSA recommendations assuming equal potency among aflatoxins [63]. The EDI values were 161.1 ng/kg bw/day for AFG1 and 0.336 ng/kg bw/day for AFB1. Accordingly, the resulting MOE values were approximately 2.5 for AFG1 and 1190 for AFB1.

According to the guidance of the European Food Safety Authority (EFSA) [64], MOE values below 10,000 indicate a potential public health concern, suggesting a possible risk associated with chronic dietary exposure to AFB1 and AFG1 in the Tunisian population. Although the MOE values indicate a potential high health risk associated with exposure to these mycotoxins, AFG1 was detected in only four samples (6.55%) and AFB1 in two samples (3.28%) of the analyzed durum wheat. This indicates that, despite their toxicological importance, the occurrence of these contaminants was limited to a small proportion of the samples. The relatively low frequency of contamination represents a positive finding; however, considering the high toxicity of AFs, continuous monitoring remains necessary to protect public health. The analyzed samples consisted of raw wheat grains. Since wheat is generally consumed after processing into flour and other wheat-based products, and since thermal processing can reduce mycotoxin levels [65], the actual exposure and risk for consumers may be lower than those estimated in this study.

To our knowledge, no previous studies from Tunisia have reported MOE values for AFG1 and AFB1 exposure through raw wheat consumption. However, Belasli et al. [66] calculated MOE values for wheat-based products in Algeria, reporting values below 10,000. While differences in food matrix and processing conditions limit direct comparison, the findings consistently indicate potential health concerns related to AFs exposure in cereal-based foods within the region. The exposure and health risk assessment was limited to Tunisian adults due to the unavailability of consumption data for other age groups. In addition, although maize showed high contamination levels for several mycotoxins, it was not included in the dietary exposure assessment because national consumption data for this cereal were unavailable. In light of the EDI and MOE results presented above, the evaluation of mycotoxin exposure through cereals remains an important issue. However, according to published studies, few investigations in Tunisia have examined mycotoxin intake from cereals or evaluated the associated health risks. Regular consumption of cereals contaminated with mycotoxins may lead to adverse health effects over time. These risks can be mitigated by educating farmers on mycotoxin prevention and control strategies and optimizing harvest and post-harvest practices. Furthermore, to protect Tunisian consumers, the establishment of regulatory limits for mycotoxins in cereals is strongly recommended.

## 3. Conclusions

The analysis of cereal samples from Tunisia revealed the occurrence of multiple mycotoxins in cereals from the Beja and Mahdia regions. Durum wheat was contaminated by AFB1, AFG1, ENA, ENA1, ENB, and ENB1, with ENB being the most frequent and abundant in Beja (70%), while ENB1 showed the highest frequency in Mahdia (22.58%). Barley was contaminated by ENA, ENA1, ENB, and ENB1, with ENB being the most frequent and abundant in Beja (66%) and Mahdia (28.57%). Maize was contaminated by OTA, ENA, ENA1, ENB, ENB1, AFB1, AFB2, and AFG2, with ENA1 being the most frequent and abundant in Beja, whereas AFB1 showed the highest frequency (35%) and abundance in Mahdia. Overall, the European Union maximum limits were exceeded for AFG1 in wheat and for aflatoxins and OTA in maize samples. However, it is important to highlight that the occurrence of these mycotoxins in the analyzed samples was low, with a prevalence below 18%. Co-occurrence of mycotoxins was detected in 60% of the positive samples, with each sample containing between two and six mycotoxins, including both regulated and emerging mycotoxins. The contamination and co-contamination of cereals, particularly by ENs, represent a potential health concern, as these cereals are traditional staple foods widely consumed by the Tunisian population. A risk assessment was performed for mycotoxins detected in wheat and barley, revealing high EDI values for AFG1 and ENs through the consumption of these cereals among Tunisian adults. As part of this assessment, risk characterization was performed for AFB1 and AFG1 by calculating the margin of exposure (MOE). The obtained MOE values were below 10,000 (MOE = 1190 for AFB1 and 2.5 for AFG1), indicating a potential health concern from dietary exposure to these mycotoxins through durum wheat. Fortunately, AFB1 and AFG1 were detected in only 3.28% and 6.55% of the samples, respectively, suggesting that contamination affected only a small proportion of the wheat, providing a reassuring perspective for consumers while highlighting the need for continued monitoring of AF contamination. However, maize, which showed relatively high contamination levels, was not included in the exposure assessment due to the lack of consumption data.

To reduce fungal growth and mycotoxin contamination in cereals and protect human health, several practical measures should be applied. These include harvesting cereals at the appropriate time, drying grains rapidly after harvest to reduce moisture content, maintaining suitable storage conditions with good ventilation, regularly monitoring temperature and humidity during storage, and cleaning storage facilities before use. In addition, training farmers on good agricultural and storage practices is essential to limit fungal contamination and mycotoxin production. In addition, stricter regulations and the establishment of maximum tolerable limits for all mycotoxins in cereals are needed in Tunisia to reduce potential health risks and economic losses. Further research on the occurrence of mycotoxins in Tunisian cereals is highly needed. Moreover, different strategies for the prevention and detoxification of mycotoxins in Tunisian foods, particularly cereals, should be explored.

## 4. Materials and Methods

### 4.1. Reagents and Chemicals

The solvents and reagents used to carry out this study are detailed below. Acetonitrile (ACN) HPLC grade was furnished by Merck (Darmstadt, Germany). The deionized water (resistivity > 18 M/cm1) was obtained in the lab using a Milli-Q SP^®^ Reagent Water System (Millipore Corporation, Bedford, MA, USA). The formic acid (95%) was delivered from Sigma Aldrich (St. Louis, MO, USA). Salts used for QuEChERS extraction include sodium chloride (NaCl) from Fisher Scientific (Geel, Belgium), octadecyl C18 sorbent from Phenomenex (Madrid, Spain) and magnesium sulfate (MgSO_4_) and anhydrous 99.5% min powder from ThermoFisher (Kandel, Germany). The nylon filters (0.45 µm pore size) were supplied by Scharlau (Barcelona, Spain). The syringe nylon filters (13 mm diameter and 0.22 µm pore size) were furnished by Membrane Solutions (Plano, TX, USA). Mycotoxin standards (AFB1, AFB2, AFG1, AFG2, OTA, ENA, ENA1, ENB and ENB1) were supplied by Sigma (St. Louis, MO, USA).

### 4.2. Regions of the Study

Two distinct agro-climatic and geographical condition regions were chosen for this study from among the regions in Tunisia that produce cereals: Mahdia, a coastal region in the Center East (latitude 35°30′17′ N; longitude 11°3′44′ E; altitude 6 m), and Beja, a continental region in the Northwest (latitude 36°43′32″ N; longitude 9°10′54″ E; altitude 148 m). These regions are characterized by semi-arid and sub-humid climates, respectively.

### 4.3. Sample Collection and Preparation

One hundred fifty-eight raw cereal grains, including durum wheat (*n* = 61), barley (*n* = 57), and maize (*n* = 40), were collected during the early post-harvest period in 2022. Durum wheat and barley samples were collected between June and July, whereas maize samples were collected between July and August. Sampling was performed from Beja (*n* = 79) and Mahdia (*n* = 79). Samples were collected from regional grain collection centers during the immediate post-harvest period when freshly harvested cereal lots from multiple local farms are received prior to storage and distribution. The distribution of samples according to cereal type and sampling region is summarized in Table 6. The samples were milled and weighed (100 g each), put in bags made from polyethylene, and then kept at −20 °C without being pre-treated in any manner. The whole collection was taken in polystyrene boxes that contained dry ice to the laboratory located at the University of Valencia in Spain, where it was analyzed. They were then stored in the laboratory at −20 °C until the analysis.

### 4.4. Mycotoxin Extraction from Raw Cereals

A QuEChERS extraction method was used to extract mycotoxins from cereals. In fact, two grams of each sample were weighed in a 50 mL conical tube; 10 mL of acidified water with 2% formic acid was then added, and the tube was shaken for 30 min in an IKA KS 260 orbital shaker. After 30 min, 10 mL of ACN had been added, and the tube was shaken for another 30 min. Then, 4 g of MgSO_4_ and 1 g of NaCl were added to the tube that contained the mixture of cereal powder and solvents; the tube was vortexed for 30 s, and the resulting mixture was centrifuged at 5000 rpm for 10 min. Then, 2 mL of the supernatant was mixed with 0.3 g of MgSO_4_ and 0.1 g of Octadecyl C18 sorbent in a 15 mL tube. The supernatant was filtered through a 13 mm/0.22 µm nylon filter (Membrane Solutions, TX, USA) after shaking and centrifuging again under the conditions described above. Finally, the extracts were stored at −20 °C prior to injection into an LC-MS/MS system.

### 4.5. UHPLC-MS/MS Determination

The mycotoxins were analyzed by UHPLC-MS/MS. The equipment was a Sciex TRIPLE QUAD 6500+ (Sciex, Concord, ON, Canada) equipped with electrospray ionization (ESI) coupled to an Agilent 1260 UHPLC system (degasser), quaternary pump and column oven with an Eksigent ULC 100 HTC-xt autosampler (Agilent Technologies, Waldbronn, Germany). The mobile phases were H_2_O with 5 mM ammonium formate and 0.1% formic acid (A) and methanol with 0.1% formic acid (B). The column employed was a BEH^®^ C18 column (1.7 μm; 100 Å, LC 50 × 2.1 mm column, Waters) maintained at a constant temperature of 30 °C. The elution gradient started with 95% of phase A, then A decreased to 0% in 13 min. Subsequently, the proportion of A increased to 5% at 15.1 min and was maintained for 3 min. Finally, the initial conditions were readjusted. The injection volume was 5 μL. The mass spectrometer was operated in positive ionization mode and Selected Reaction Multiple Monitoring (SRM) mode using a Turbo Spray IonDrive ionization source with the following conditions: curtain gas (CUR) at 30 psi, ion sputtering voltage (IS) 4.4 kV, temperature 300 °C and ion source gas (SG) 1 and 2 at 55 psi. The parameters are shown in Table 7. Throughout the analytical sequence, continuous quality control was applied, including the systematic injection of procedural blanks and matrix-matched control samples.

The methodology employed was optimized for the analysis of mycotoxins in cereals in a previous study carried out in our laboratory [67], in accordance with EU Commission Decision 2002/657 EC [68]. The recoveries obtained in this previous study, evaluated at three fortification levels (25, 50, and 100 µg/kg), ranged from 62% to 119%.

### 4.6. Assessment of Dietary Exposure to Mycotoxins and Health Risk Characterization

The dietary exposure to mycotoxins is considered a key component in mycotoxin risk assessment. To evaluate the dietary exposure of Tunisian adults through the consumption of wheat and barley, the Estimated Daily Intake (EDI) was calculated. The EDI, expressed in ng/kg bw/day, is determined based on the contamination level of the mycotoxin in the analyzed food, the daily consumption of that food by the population, and the average body weight of the individuals. The EDI was determined according to the formula presented below [28]:

(1) $$EDI=\frac{MC\times ADI}{BW}$$

In this context, the mean concentrations of mycotoxins (MC) represent the analyzed wheat and barley samples (µg/kg), ADI represents the average daily intake of wheat or barley by the Tunisian (g/person/day) and BW represents the average body weight, assumed to be 70 kg for Tunisian adults [39].

To estimate dietary exposure to AFB1 through the consumption of durum wheat, the two samples with detectable AFB1 concentrations below the LOQ were assigned the LOQ value (1 µg/kg), while samples with no detectable AFB1 were assigned zero before calculating the mean concentration across all analyzed wheat samples.

Health risk characterization was conducted selectively based on the availability of toxicological reference values. For AFB1 and AFG1, risk characterization was performed using the margin of exposure (MOE) approach, as these mycotoxins are genotoxic and carcinogenic, and therefore no safe intake level can be established. In contrast, risk characterization for ENs was not performed due to the absence of tolerable daily intake (TDI) values or other health-based guidance limits. The MOE for AFB1 and AFG1 was calculated using the following formula:

(2) $$MOE=\frac{{BMDL}_{10}}{EDI}$$

In this approach, the benchmark dose lower confidence limit (BMDL) represents the statistically derived lower confidence limit of the dose associated with a predefined toxicological effect, corresponding to no more than a 10% increase in cancer incidence (BMDL_10_) [64]. The BMDL is obtained through quantitative dose–response modeling based on experimental or epidemiological data and serves as a key reference value in toxicological risk assessment, expressed in µg/kg bw/day. The EDI is expressed in ng/kg bw/day. For AFB1 and AFG1, a BMDL_10_ value of 0.4 μg/kg body weight/day was applied [63]. An MOE value ≥ 10,000 indicates a low level of concern, whereas an MOE < 10,000 suggests a potential health risk [64].

### 4.7. Statistical Analysis

A two-way ANOVA was employed to assess the impact of cereal type and geographic region on mycotoxin concentrations. Statistical analyses were conducted using SPSS (v. 10.0; SPSS Inc., Chicago, IL, USA, 2000), with significance defined as *p* < 0.05.

## Figures and Tables

**Table 1:** Prevalence of mycotoxins in different Tunisian cereal samples.

Type of Raw Cereal (*n*)	Detected Mycotoxins	Number of Positive Samples	Frequency (%)	Minimum Concentration (µg/kg)	Maximum Concentration (µg/kg)	Mean Concentration ^1^ (µg/kg)	Mean Concentration ^2^ (µg/kg)
Durum wheat (61)	AFB1	2	3.28	<LOQ*	-	-	-
	AFG1	4	6.55	100.04	127.75	118.08	15.74
	ENA	10	16.39	60.53	131.75	73.32	12.02
	ENA1	19	31.14	45.63	350.33	75.88	24.12
	ENB	28	45.90	0.44	1385.83	152.36	69.93
	ENB1	20	32.78	15.06	155.81	50.58	16.58
Barley (57)	ENA	4	7	60.98	81.66	68.88	4.83
	ENA1	7	12.28	46.16	167.52	74.38	9.13
	ENB	27	47.37	16.75	266.14	41.42	14.93
	ENB1	9	15.79	12.33	79.17	25.20	3.98
Maize (40)	AFB1	7	17.50	40.94	1769.89	745.83	261.04
	AFB2	4	10	16.89	2236.82	575.18	115.04
	AFG2	1	2.5	593.17	593.17	593.17	29.66
	OTA	2	5	92.88	99.93	96.40	9.64
	ENA	9	22.5	60.88	93.45	66.28	15.05
	ENA1	9	22.5	45.47	58.25	47.81	10.76
	ENB	7	17.50	17.46	1113.37	174.42	30.52
	ENB1	4	10	15.16	17.95	16.33	1.63

^1^ Mean concentration of mycotoxin in positive samples; ^2^ mean concentration of mycotoxin in all samples; *n*: total number of collected samples; LOQ*: limit of quantification.

**Table 2:** Prevalence of mycotoxins in different cereal samples collected from two different regions in Tunisia.

Raw Cereal Type	Region (*n*)	Detected Mycotoxin	Number of Positive Samples	Frequency (%)	Minimum Concentration (µg/kg)	Maximum Concentration (µg/kg)	Mean *p* Concentration ^1^ (µg/kg)	Mean Concentration ^2^ (µg/kg)
Wheat	Beja (30)	AFB1	2	7	<LOQ	-	-	-
		AFG1	4	13	100.44	127.75	116.22	15.74
		ENA	8	27	60.53	131.75	76.28	20.34
		ENA1	13	43	46.03	350.33	91.01	39.44
		ENB	21	70	0.44	1385.83	214.99	138.15
		ENB1	14	47	16.07	155.81	63.89	29.82
	Mahdia (31)	ENA	2	6.45	60.86	62.10	61.48	3.97
		ENA1	6	19.35	45.63	55.72	47.99	9.29
		ENB	7	22.58	11.23	19.56	18.21	3.91
		ENB1	6	19.35	15.06	31.56	19.53	3.78
Barley	Beja (29)	ENA	1	3	60.98	60.98	60.98	2.10
		ENA1	3	10	46.16	46.74	46.50	4.81
		ENB	19	66	16.94	23.7	18.86	12.36
		ENB1	5	17	16.2	18.86	17.29	2.98
	Mahdia (28)	ENA	3	10.71	61.76	81.66	71.51	7.66
		ENA1	4	14.28	55.64	167.52	95.29	13.61
		ENB	8	28.57	16.75	266.14	61.59	17.60
		ENB1	4	14.28	12.33	79.17	35.10	5.01
Maize	Beja (20)	OTA	2	10	92.88	99.93	96.40	9.64
		ENA	2	10	61.44	61.70	61.57	6.16
		ENA1	3	15	45.47	47.29	46.45	6.97
		ENB	2	10	18.11	18.13	18.13	1.81
		ENB1	1	5	15.16	15.16	15.16	0.76
	Mahdia (20)	AFB1	7	35	40.94	1769.89	745.83	261.04
		AFB2	4	20	16.89	2236.82	575.18	115.04
		AFG2	1	5	593.17	593.17	593.17	29.66
		ENA	7	35	60.88	93.45	68.39	23.94
		ENA1	6	30	45.54	58.25	48.49	14.55
		ENB	5	25	17.46	1113.37	236.94	59.23
		ENB1	3	15	15.46	17.95	16.72	2.51

^1^ Mean concentration of mycotoxin in positive samples; ^2^ mean concentration of mycotoxin in all samples; *n*: total number of collected samples.

**Table 3:** Meteorological conditions during the 2022 sampling period in Tunisia.

Region	Period of Collection	Range of Temperatures (Average) °C	Range of Humidity (Average) %
Littoral region (Mahdia)	June–July	24.2–34.8	-
		(30)	(62)
	July–August	25.9–34.8	62–69
		(31)	(66)
Continental region (Beja)	June–July	23.1–39.9	48–52
		(32.5)	(50)
	July–August	23.2–39.9	52–63
		(32)	(58)

Data from the National Institute of Meteorology (Tunisia).

**Table 4:** Profile of mycotoxin co-occurrence in different cereal types collected from two Tunisian regions.

Region	Raw Cereal Type (*n*)	Contamination (Number of Positive Samples, Frequency)				
		By 2 Mycotoxins	By 3 Mycotoxins	By 4 Mycotoxins	By 5 Mycotoxins	By 6 Mycotoxins
Continental Region (Beja) *n* = 79	Wheat (*n* = 30)	ENB + ENB1 or ENA + ENB (2, 6.70%)	ENA1 + ENB + ENB1 (5, 16.70%)	ENA + ENA1 + ENB + ENB1 (5, 16.7%)	AFG1 + ENA + ENA1 + ENB + ENB1 (1, 3.33%)	AFB1 + AFG1 + ENA + ENA1 + ENB + ENB1 (1, 6.70%)
	Barley (*n* = 29)	ENB + ENB1 (2, 6.90%)	ENA1 + ENB + ENB1 (2, 6.90%)	ENA + ENA1 + ENB + ENB1 (1, 3.45%)	-	-
	Maize (*n* = 20)	-	ENA + ENA1 + OTA (1, 5%)	ENA + ENA1 + ENB + ENB1 (1, 5%)	-	-
Littoral region (Mahdia) *n* = 79	Wheat (*n* = 31)	-	ENA1 + ENB + ENB1 (5, 16.13%)	ENA + ENA1 + ENB + ENB1 (1, 3.22%)	-	-
	Barley (*n* = 28)	ENB + ENB1 (1, 3.58%)	-	ENA + ENA1 + ENB + ENB1 (3, 10.71%)	-	-
	Maize (*n* = 20)	AFB2 + AFG2 or ENA + ENA1 or ENA + ENB (5, 25%)	ENA + ENA1 + ENB1 (1, 5%)	ENA + ENA1 + ENB + ENB1 (1, 5%)	AFB1 + AFB2 + ENA + ENA1 + ENB (1, 5%)	AFB1 + AFB2+ ENA + ENA1 + ENB + ENB1 (1, 5%)
Total (*n* = 158)		10	14	12	2	2

*n*: total number of collected samples.

**Table 5:** Estimated Daily Intake (EDI) and Margin of Exposure (MOE) for mycotoxins detected in cereal samples collected in Tunisia in 2022.

Type of Cereal	Detected Mycotoxin	Mean Concentration ^1^ (µg/kg)	EDI (ng/kg bw/Day)	MEO
	AFB1	0.0328 ^2^	0.336	1190
Raw Durum Wheat	AFG1	15.74	161.10	2.5
	ENA	12.02	123.00	-
	ENA1	24.12	247.00	-
	ENB	69.93	716.20	-
	ENB1	16.58	169.80	-
Raw Barley	ENA	4.83	8.50	-
	ENA1	9.13	16.09	-
	ENB	14.93	26.30	-
	ENB1	3.98	7.00	-

^1^ Mean concentration of mycotoxin in all samples; ^2^ concentrations of AFB1 below the LOQ were substituted with the LOQ value for exposure assessment calculations.

**Table 6:** Sampling details.

Raw Cereal Samples (*n* = 158)	Regions	
	Mahdia (Coastal Region) *n* = 79	Beja (Continental Region) *n* = 79
Durum wheat (*n* = 61)	31	30
Barley (*n* = 57)	28	29
Maize (*n* = 40)	20	20

*n*: total number of collected samples.

**Table 7:** Optimized mass spectrometry parameters.

						Quantification Ion (Q)			Confirmation Ion (q)		
Mycotoxin	t_R_ (min)	LOD (µg/Kg)	LOQ (µg/Kg)	DP	Precursor Ion (m/z)	CE	Product Ion (m/z)	CXP	CE	Precursor Ion (m/z)	CXP
AFB1	8.1	0.3	1	106	313.1	33	285.2	16	91	128.1	10
AFB2	7.7	0.3	1	96	315.1	37	287.2	18	43	259.2	18
AFG1	7.5	0.3	1	86	329.1	39	243.1	14	59	200	12
AFG2	7.2	1	3.3	111	331.1	35	313.2	18	43	245.2	14
OTA	10.1	1	3.3	91	404	37	239	16	105	102	14
ENA	12.6	0.3	1	106	699.4	43	210.1	12	47	228	18
ENA1	12.4	0.3	1	96	685.4	46	210.1	8	49	228.2	20
ENB	12	0.3	1	81	657.5	45	196.3	18	47	214.1	18
ENB1	12.2	0.3	1	111	671.4	43	196	12	41	210	12

t_R_: retention time; LOD: limit of detection; LOQ: limit of quantification; DP: declustering potential; CE: collision energy; CXP: collision cell exit potential; m/z: mass-to-charge ratio; Q: quantification ion; q: confirmation ion.

## Data Availability

The original contributions presented in this study are included in the article. Further inquiries can be directed to the corresponding authors.

## Abbreviations

The following abbreviations are used in this manuscript:

ACN	Acetonitrile
AFB1	Aflatoxin B1
AFB2	Aflatoxin B2
AFG1	Aflatoxin G1
AFG2	Aflatoxin G2
AFs	Aflatoxins
BMDL_10_	Benchmark Dose Lower Confidence Limit for a 10% response
CUR	Curtain Gas
CE	Collision Energy
CXP	Collision Cell Exit Potential
DON	Deoxynivalenol
DP	Declustering Potential
EDI	Estimated Daily Intake
ESI	Electrospray Ionization
ENA	Enniatin A
ENA1	Enniatin A1
ENB	Enniatin B
ENB1	Enniatin B1
ENs	Enniatins
EU	European Union
FMs	Fumonisins
H_2_O	Water
HPLC	High-performance liquid chromatography
LC-MS/MS	Liquid chromatography–tandem mass spectrometry
LOQ	Limit of Quantification
LOD	Limit of Detection
MgSO4	Magnesium Sulfate
MOE	Margin of Exposure
MPL	Maximum Permitted Level
NaCl	Sodium Chloride
OTA	Ochratoxin A
PAT	Patulin
Q	Quantification Ion
q	Confirmation Ion
SRM	Selected Reaction Monitoring
T-2	T-2 toxin
TDI	Tolerable Daily Intake
tR	Retention Time
TRC	Trichothecenes
UHPLC–MS/MS	Ultra-high-performance liquid chromatography coupled to tandem mass spectrometry
UP	Ultrahigh Pressure
ZEN	Zearalenone
